# Mitochondrial Disorders with Significant Ophthalmic Manifestations

**DOI:** 10.4103/0974-9233.51998

**Published:** 2008

**Authors:** Mona Al-Enezi, Hanan Al-Saleh, Murad Nasser

**Affiliations:** From the Department of Ophthalmology, Mohammed Abdul Rahman Al-Bahar Eye Center, Ibn Sina Hospital, Kuwait

**Keywords:** Mitochondrial disorder genetics, variable manifestations, diagnosis

## Abstract

Mitochondrial diseases are a clinically hetyerogenous group of disorders. They can be caused by mutations of nuclear or mitochondrial DNA (mtDNA). Some affect a single organ, but many involve multiple organ systems and often present with prominent neurologic and myopathic features. The eye is frequently affected, along with muscles and brain, but multisystem disease is common. Ophthalmic manifestations include cataract, retinopathy, optic atrophy, cortical visual loss, ptosis and ophthalmoplegia. Kearns-Sayre Syndrome (KSS), Mitochondrial Encephalopathy, Lactic Acidosis Stroke (MELAS), Myoclonic Epilepsy and Ragged Red Fiber myopathy (MERRF) and Lebers Hereditary Optic Neuropathy (LHON) are well known clinical entities that are secondary to mtDNA abnormalities, which has ophthalmic manifestations. Mitochondrial Dysfunction should be considered in the differential diagnosis of progressive multisystem disorder and specifically if there is associated neuro-ophthalmic manifestations, which may be the presenting symptom of these disorders.

Structurally mitochondria have four components. The outer membrane space, the inner membrane, the intermembrane space and the matrix. They perform numerous tasks, such as pyruvate oxidation, the Krebs cycle, and metabolism of amino acids, fatty acids and steroids, but the most crucial is probably the generation of energy as adenosine triphosphate (ATP) by means of the electron transport chain and the oxidative phosphorylation system (the respiratory chain).

Mitochondria are the only organelles of the cell besides the nucleolus that contain their own. DNA (called mtDNA) own machinery for synthesizing RNA and proteins. There are hundreds or thousands of mitochondria per cell.^1^,^2^

Defects in any of the numerous mitochondrial pathways can cause mitochondrial diseases, but we will, confine our discussion to disorders secondary to defect in the respiratory chain with significant ophthalmic manifestations.

The mitochondrial respiratory chain is the essential final common pathway for aerobic metabolism. Tissues and organs that are highly dependent upon aerobic metabolism are preferentially involved in mitochondrial disorders, such as peripheral nervous system, central nervous system, endocrine glands, heart, ears, eyes, gastrointestinal tract, kidneys, bone marrow and dermis.

Over 70 different polypeptides interact on the inner mitochondrial membrane to form the respiratory chain. The vast majority of subunits are synthesized within the cytosol from nuclear gene transcripts, but 13 essential subunits are encoded by mitochondrial DNA (mtDNA).^1^

Although clinically distinct, most mt DNA-related diseases share the features of lactic acidosis and massive mitochondrial proliferation in muscles (resulting in ragged-red fibers). In muscle biopsy specimens, the mutant mtDNA accumulate preferentially in ragged red fibers. Ragged red fibers are typically negative for cytochrome oxidase activity.^1^

The fact that the respiratory chain is under dual genetic control makes these disorders particularly fascinating because they involve both mendelian and mitochondrial genetics.^3^ Moreover these disorders are not as rare as commonly believed, their estimated prevalence of 10 to 15 cases per 100,000 persons is similar to that of better known disease as muscular dystrophies.^1^

## Mitochondrial Genetics

Mitochondrial disorders may be caused by defects of nuclear DNA or mtDNA. Nuclear gene defects may be inherited in an autosomal recessive manner or an autosomal dominant manner.^1^,^2^,^3^,^4^ Mitochondrial DNA are transmitted by maternal inheritance. All mitochondria (and all mtDNAs) in the Zygote are derived from the ovum. Therefore, a mother carrying a mtDNA mutation passes it to all her children, but only her daughters will transmit it to their progeny. The father of a proband is not at risk of having the disease, but the mother usually has the mitochondrial mutation and may and may not have the symptoms. A male does not transmit the mtDNA to his offsprings. A female harboring a mtDNA point mutation may transmit a variable amounts of mutant mtDNA to her offsprings, resulting in considerable clinical variability among siblings within the same family.^4^,^5^

There is no straight forward relation between the site of the mutation and the clinical phenotype, even with a mutation in a single gene.^1^,^6^ For example, mutations in the mtRNA gene are usually associated with mitochondrial encephalomyopathy, lactic acidosis and stroke like episodes (MELAS) syndrome, but they cause other syndromes as well. Conversely mutations in different genes can cause the same syndrome, MELAS; again is a prime example.^1^,^6^

There are exceptions virtually all patients who have the myoclouns epilepsy with ragged red fiber (MERRF) syndrome have mutations in the mtRNA gene, all patients with leber's hereditary optic neuropathy have mutations in ND genes, and most mutations in the cytochrome gene cause exercise intolerance.^1^

Most mtDNA mutations are certain mtDNA point mutations. Most mtDNA mutations are heteroplasmic, which means that they only affect a proportion of copies of mtDNA. Clinical syndromes can usually be caused by several different mutations and conversely, the same mutation can present in different ways depending on the level of heteroplasmy.^1^,^6^

## Heteroplasmy and The Threshold Effect

There are thousands of mtDNA molecules in each cell, and in general, pathogenic mutations of mtDNA are present in some but not all of these genomes. As a result, cells and tissues harbor both normal (wild type) and mutant mtDNA, a situation known as heteroplasmy.^1^,^4^,^7^ Heteroplasmy can also exist at the organelle level a single mitochondria can harbor both normal and mutant mtDNA, in normal subjects all mtDNAs are identical (homoplasm). A minimal number of mutant mtDNAs must be present before oxidative dysfunction occurs and clinical signs become apparent, this is the threshold effect. The threshold for disease is lower in tissues that are highly dependent on oxidative metabolism, such as brain, heart, skeletal muscles retina, renal tubules and endocrine glands. These tissues will therefore be especially vulnerable to the effects of pathogenic mutations in mtDNA.

As a result of heretroplasmy and the threshold effect, different tissues harboring the same mtDNA mutation may be affected to different degrees, thus explaining the frequent occurrence of oligosymptomatic or asymptomatic carriers of the mutation within a family. Selective organ involvement can also occur, presumably as a result of skewed heteroplasmy (i.e., disproportionately high levels of the mutation in a given tissue) as in mitochondrial diabetes, mitochondrial myopathies and mitochondrial deafness.^1^ The pathogenesis of these disorders is unclear although impaired production of ATP most likely has a central role.

## Mitotic Segregation

The random distribution of organelles at the time of cell division can change the proportion of mutant mtDNA, received by daughter cells, if and when the pathogenic threshold in a previously unaffected tissue is surpassed, the phenonotype can also change. This explains the age related and even tissue related variability of clinical features frequently observed in mtDNA related disorders.

For all mtDNA mutations clinical expression depends on three factors:

1. Heteroplasmy, the relative abundance of mutant mtDNA.
2. Tissue distribution of mutant mtDNAs.
3. Threshold effect. The vulnerability of each tissue to impaired oxidative metabolism.^4^

## Neuro-ophthalmic Manifestations of Mitochondrial Disease

The four most common neruophthalmic abnormalities seen in mitochondrial disorders are bilateral optic neuropathy, ophthalmoplegia and ptosis, pigmentary retinopathy and retro chasmal visual loss.

1. Bilateral optic atrophy as in LHON.
2. chronic progressive ophthalmoplegia (CPEO): It is the most common ocular manifestation of mitochondrial myopathies.^8^ It is a group of clinical findings characterized by slowly progressive bilateral ocular immobility associated with ptosis. Ophthalmoplegia may be isolated or associated with other neurological or systemic abnormalities, such as bulbar and limb myopathies, deafness, ataxia, spasticity, peripheral neuropathy, gastrointestinal myopathy and neuropathy, vestibulbor dysfunction, dementia, episodic encephalopathy or coma and calcification of the basal ganglia. Associated ocular features include optic atrophy, pigmentary retinopathy, corneal changes and cataracts.^9^ Kearns-sayre (KSS) syndrome is a subset of CPEO.
3. Pigmentary retinopathy: Pigmentary changes in the retina may occur in patients with mitochondrial disease. The most common appearance is that of a salt and pepper retinopathy, which typically becomes more prominent with age.^6^ There may be profound macular involvement, vascular attenuation is common, degeneration of the retinal pigment epithelium and abnormalities of the rods and cones usually occur. Visual loss occurs in approximately 50 percent of patients and is usually mild. Pigmentary retinopathy is one of the diagnostic criteria in KSS. RP degeneration can be seen in patients with CPEO and no other neruologic or systemic abnormalities. Pigmentary retinopathy can also occur in patient with mitochondrial disease who does not have CPEO such as MELAS and Leigh syndromes.
4. (Retro-chiasmal) Visual Loss: Patients with mitochondrial disease may have visual loss not ascribable to optic nerve or retinal dysfunctions, but rather a reflection of the disruption of the retro-chiasmal visual pathways, thereby resulting in homonymous hemianopic defects or cortical blindness. The mitochondrial disease most consistently associated with retro-chiasmalvisual loss is MELAS.

## Mitochondrial Encephalopathy, Lactic Acidosis and Stroke (MELAS)

Mitochondrial encephalopathy, lactic acidosis and stroke like episodes is a multisystem disorder with onset typically occurring in childhood. Early psychomotor development is usually normal, but short stature is common.^1^,^7^ First onset of symptoms is frequently between ages of two and ten years.

The most common initial symptoms are generalized tonic-clonic seizures, recurrent headaches, anorexia and recurrent vomiting. Seizures are often associated with stroke like episodes of transient hemi paresis or cortical blindness.^7^,^10^

Migrainous headaches occur in the majority of affected individuals and in maternal relatives. Ocular manifestations include progressive external ophthalmoplegia “salt and pepper” pigmentary retinopathy, optic atrophy and macular pattern dystrophy.

Sensorineural hearing loss is common. Less common symptoms include ataxia, episodic coma, diabetes mellitus, nephropathy and gastrointestinal dysmotility. Several studies suggested that cardiac involvement frequently occurs in patients with MELAS. Ito et al reviewed 21 patients with MELAS and reported that 8 patients showed left ventricular hypertrophy. Rynichiro et al reported five patients with MELAS where 2 patients showed symmetrical hypertrophy of the left ventricles, one also had wall motion abnormalities. They concluded that left ventricular abnormalities is a characteristic clinical features in the heart patients with MELAS.^11^

The cumulative residual effects of the stroke like episodes gradually impair motor abilities vision and mentation, often by adolescence or young adulthood.

It is a maternally inherited disease, where 80 percent of patients with MELAS syndrome carry the 3243A—G mtDNA mutation. The severity for problems associated with this mutation depends on the degree of heteroplasmy.^1^ Patients with lower levels of mutant mtDNA may present with diabetes and deafness, cardiomyopathy or myopathy.

Diagnosis of MELAS is based on a combination of clinical findings and molecular genetics and the finding of elevated lactate and pyruvate concentration at rest and becomes excessively elevated after exercise. And the findings of ragged red fibers (RRF) on muscle biopsy.^10^,^12^

## Pearsons Syndrome and KSS

These syndromes are associated with mtDNA rearrangements (large deletions or duplications). They are always heterogenous and the concentration of the rearranged mtDNA in different tissues determines the age and manner of presentation: not all conform to the syndromes.^6^

Infants with Pearson Syndrome have sideroblastic anemia, pancytopenia, pancreatic exocrine insufficiency and liver disease.^5^ Renal tubular dysfunction, diabetes mellitus and intestinal atrophy may also be seen in these patients. Hematological problems generally improve but features of Kearns-Sayre syndrome emerge in patients who survive long enough.

Kearns-Sayre syndrome is a progressive external ophthalmoplegia^13^ (PEO) and pigmentary retinopathy with onset before 20 years of age with at least one of heart block, cerebellar syndrome or high CSF proteins. Ptosis is the commonest complaint, patients seldom suffer diplopia even when there is sever decrease in eye movement because of gradual progression. Other features include hearing loss, dementia, cardiomyopathy and endocrine disorders.^2^ The most mildly effected patients with mtDNA rearrangements present as adults isolated ophthalmoplegia and ptosis.

The ophthalmoplegia associated with mtDNA rearrangements appears to result from muscle abnormalities; ptosis is common, as is exotropia, often without diplopia presumably because of the gradual onset. Most patients with Kearns-Sayre syndrome develop a “salt and pepper” fundus. Visual acuity tends not to be severely impaired, unless there is optic atrophy. Other patients have generalized loss of the retinal pigment epithelium.

### Ophthalmic Features

*Progressive external ophthalmoplegia (CPEO) and ptosis. Retinal dystrophy.* Most patients retain good visual function with mild but probably progressive cone-rod dystrophy and a “salt and pepper” appearance with regions of increased and decreased pigmentation particularly in the equatorial fundus. Other cases develop severe receptor loss, blindness and no recordable electroretinogram (ERG): these may either have a typical retinitis pigmentosa fundus or severe retinal pigment epithelial atrophy which may mimic choroideremia.

*Corneal Clouding:* Rarely KSS may present with corneal clouding associated with either congenital glaucoma or corneal dystrophy. Structural changes in endothelium and Descemet's membrane have been reported with corneal edema may be due to reduced pump action of corneal mitochondria. Rarely corneal changes may precede systemic signs by several years.^6^,^14^

*Optic Neuritis:* Patients with Pearsons syndrome and KSS amy also suffer from optic neuritis.

*Muscular Dystrophy:* Studies on muscle biopsies generally show that a proportion of fibers have cytochrome-c-oxidase deficiency and there may also be “ragged red fibers”. Pearson syndrome and Kearns-Sayre syndrome are almost always sporadic and, if an unaffected mother has affected child, the recurrence risk is low. Pearson syndrome has been reported in children of women with Kearns-Sayre syndrome.

## Leber's Hereditary Optic Neuropathy

Since the late 1980s, LHON has received notoriety as a maternally inherited disease linked to abnormalities in mitochondrial DNA.

Men are affected with visual loss more frequently than women, with a male predominance of approximately 80 to 90 percent in most pedigrees. A minimum of 25 percent of men and 5 percent of women at risk for LHON experience visual loss.^2^,^5^ The onset of visual loss typically occurs between the ages of 15 and 35 years, but otherwise classic LHON has been reported in many individuals both younger and older, with a range of age at onset from 2 to 80 years.^16^,^17^,^18^ Visual loss typically begins painlessly and centrally in one eye. The second eye is usually affected weeks to months later. Reports of simultaneous onset are numerous and likely reflect both instances of true bilateral coincidence and those in which initial visual loss in the first eye went unrecognized. More than 97 percent of patients will have second eye involvement within 1 year.^17^,^18^ The duration of progression to visual loss in each eye also varies and may be difficult to document accurately. Usually, the course is acute or subacute, with deterioration of visual function stabilizing after months.

Visual acuities in LHON patients at the point of maximum visual loss range from no light perception to 20/20^16^,^17^,^18^ with most patients deteriorating to acuities worse than 20/200. Color vision is severely affected, often early in the course, but rarely before considerable acuity loss. Pupillary light responses may be relatively preserved when compared with the responses in patients with optic neuropathies from other causes (sometimes manifesting as a smaller or absent relative afferent pupillary detect in clinically unilateral cases).^1^ Visual field defects are typically central or cecocentral.

**Figure 1:**
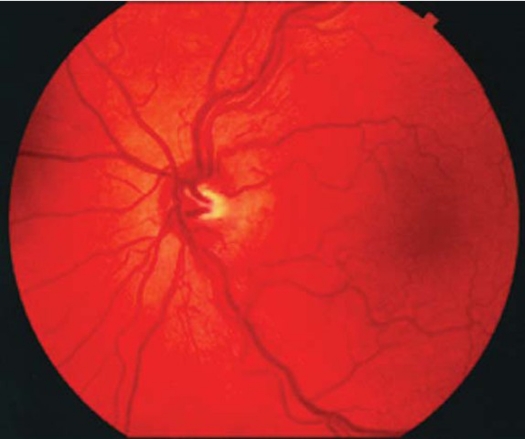
Fundus photograph of the left eye showing swollen disc with some telangeictatic vessels (Dr R. Behbehanie patient with permission).

Funduscopic abnormalities, especially during the acute phase of visual loss, include hyperemia of the optic nerve head with obscuration of the disc margins, dilation and tortuosity of posterior pole vasculature, (Figure 1) and, less commonly, retinal and disc hemorrhages, macular exudates, and retinal striations.^19^ A triad of signs is believed to be pathognomonic for LHON: circumpapillary telangiectic microangiopathy, swelling of the nerve fiber layer around the disc (pseudoedema, Figure 1), and absence of leakage from the disc or papillary region on fluorescein angiography (distinguishing the LHON nerve head from truly oedematous discs).^9^ These findings can be found not only in patients in the acute phase of visual loss, but also in presymptomatic eyes, as well as in the eyes of asymptomatic maternal relatives.^16^ Indeed, having abnormalities of the peripapilary nerve fiber layer does not necessarily predict visual loss. Furthermore, some patients with LHON never exhibit characteristic ophthalmoscopic appearance, even examined at the time of acute visual loss.^17^,^18^ Hence, the “classic” LHON ophthalmoscopic appearance may be helpful in suggesting the diagnosis if recognized in patients or their maternal relatives, but its absence—even during the period of acute visual loss—does not exclude the diagnosis of LHON. As the disease progresses, the telangiectatic vessels disappear and the pseudoedema of the disc resolves. Perhaps because of the initial hyperemia, the optic discs of patients with LHON may not appear pale for some time. This feature, coupled with the relative preserved pupillary responses and the lack of pain, has lead to the misdiagnosis of nonorganic visual loss in some LHON patients. Eventually, however, optic atrophy with nerve fiber layer dropout most pronounced in the papillomacular bundle supervenes. Nonglaucomatous cupping of the optic discs may also be seen in patients with symptomatic LHON.

In most patients with LHON, visual loss remains profound and permanent. However, recovery of ever excellent central vision may occur years after visual deterioration.^17^ It may take the form of a gradual clearing of central vision or restricted to a few central degrees, resulting in a small island of vision within a large central scotoma. Recovery is usually bilateral but may be unilateral. Those patients whose vision improves most substantially appear to have a lower mean age at the time of initial visual loss, but the specific mitochondrial DNA genotype is even more predictive. Recurrences of visual failure are extremely rare among those patient, both with and without, visual recovery.^20^,^21^

**Figure 2:**
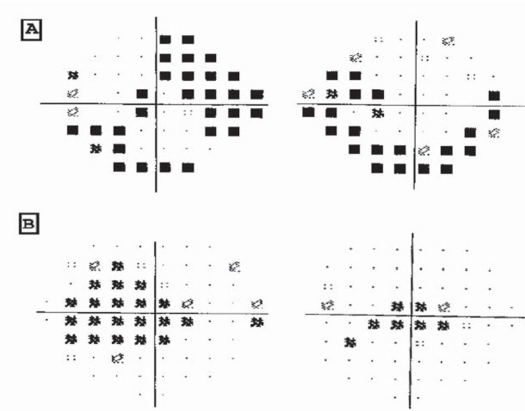
Static perimetry using **A:** the 24-2 program shows an inferior arcuate defect in the right eye a supranasal and an inferior defect in the left eye; **B:** 10-2 program shows bilateral central defects.

Investigations for LHON are blood testing for mtDNA mutation, visual field testing (Figure 2), visual evoked potential, flourescien angiography, and optical coherence tomography. In a study by Barboni et al, which included 38 LHON patients and 75 age matched control group. In this study, the thickness of the retinal nerve fiber layer was measured using Stratus OCT.^22^,^23^ They found that eyes with early LHON (where the duration of the disease was less than 6 months) showed a thicker RNFL whereas, eyes with atrophic LHON (when the duration was longer than 6 months) RNFL was thinner. The temporal fibers are the first and most severely affected.

All pedigrees, clinically designated as LHON, have a maternal inheritance pattern.

Three point mutations in the mitochondrial DNA (mtDNA), the so-called “primary” LHON mutations, are believed to account for at least 90 percent of cases of LHON worldwide.^24^ They are located at mtDNA positions 11778 (69 percent of cases), 3460 (13 percent of cases), and 14484 (14 percent of cases).^19^,^20^ Several other mtDNA mutations may be “primary” but account individually for only a few pedigrees worldwide.^19^,^20^ Screening for LHON in a patient with visual loss from optic neuropathy should begin with the three primary mutations.^5^,^15^ In those primary mutation-negative patients in whom suspicion remains high, testing for the other mtDNA mutations associated with LHON is probably warranted.

Genetic analysis allows a broader view of what constitutes the clinical profile of LHON.^1^ Most striking are the number of patients without a family history of visual loss. Some of these singleton cases are women, some outside the typical age range for LHON, some without the classic ophthalmoscopic appearance. Clearly, the diagnosis of LHON should be considered in any case of unexplained bilateral optic neuropathy, regardless of age of onset, gender, family history, or funduscopic appearance.^19^

Among 136 patients with the 11778 mutation, only 4 percent reported spontaneous recovery, compared with 37 to 65 of 14484 mutation patients.^20^,^21^ Furthermore, the ultimate visual acuities in patients with the 14484 mutation are considerably better than those with the 11778 and 3460 mutations^20^,^21^

A mtDNA mutation will be present in all maternally related family members of patients with LHON, even though many will never become symptomatic. Hence the presence of a mtDNA mutation may be necessary for phenotype expression. It may not be sufficient. Nuclear-encoded factors modifying mtDNA expression, mtDNA products, or mitochondrial metabolism may influence phenotype expression of LHON. Although most studies have not been able to confirm X-linkage as an explanation of the male predominance of visual loss in LHON, the X-linkage hypothesis may still be viable.^21^ Environmental factors, both internal and external, may play a role. Systemic illnesses, nutritional deficiencies, medications, or toxins that stress or directly or indirectly inhibit mitochondrial metabolism have been suggested to initiate or increase phenotype expression of the disease. Some reports suggest a possible role for tobacco^25^ and excessive alcohol use as precipitants of visual loss. Other agents known to be toxic to the optic nerve, such as ethambutol^19^ or to mitochondrial function, such as antiretroviral therapy, may have a heightened toxicity in patients with the LHON mutations and already compromised mitochondrial function.

## Diagnosis of a Mitochondrial Disorder

Mitochondrial dysfunction should be considered in the differential diagnosis of any progressive multisystem disorder. The diagnosis is most challenging when only one symptom is present and easier when two or more seemingly unrelated symptoms are present, involving more than one organ system. The investigation can be relatively straight forward if a person has a recognizable phenotype and if it is possible to identify a known pathogenic mtDNA mutation.^4^,^7^

- When the presentation is classic for a maternally inherited mitochondrial syndrome, such as MELAS, MERRF, or Leber hereditary optic neuropathy, appropriate mtDNA studies should be obtained first.
- When the clinical picture is classic for a nuclear DNA-inherited syndrome, the clinician should proceed with molecular genetic studies.
- When the clinical picture is nonspecific but highly suggestive of a mitochondria disorder, the clinician should start with measurement of plasma or CSF lactic acid concentration, ketone bodies, plasma acylcarnitines, and urinary organic acids. If these studies are abnormal, the clinician should proceed with muscle biopsy and assessment of the respiratory chain enzymes. Normal plasma or CSF lactic acid concentration does not exclude the presence of a mitochondrial disorder.
